# Cooperative Role of MAPK Pathways in the Interaction of *Candida albicans* with the Host Epithelium

**DOI:** 10.3390/microorganisms8010048

**Published:** 2019-12-25

**Authors:** Inês Correia, Daniel Prieto, Elvira Román, Duncan Wilson, Bernhard Hube, Rebeca Alonso-Monge, Jesús Pla

**Affiliations:** 1 iBiMED-Institute of Biomedicine, Department of Medical Sciences, Agra do Crasto, University of Aveiro, 3810-193 Aveiro, Portugal; inescorreia351@gmail.com; 2 Departamento de Microbiología y Parasitología-IRYCIS, Facultad de Farmacia, Universidad Complutense de Madrid, Plaza de Ramón y Cajal s/n, E-28040 Madrid, Spain; adprieto@farm.ucm.es (D.P.); elvirarg@farm.ucm.es (E.R.); realonso@farm.ucm.es (R.A.-M.); 3 Medical Research Council Centre for Medical Mycology, School of Biosciences, University of Exeter, Exeter EX4 4QD, UK; Duncan.Wilson@exeter.ac.uk; 4 Department of Microbial Pathogenicity Mechanisms, Leibniz Institute for Natural Product Research and Infection Biology-Hans-Knoell-Institute, Beutenbergstraße 11A, 07745 Jena, Germany; Bernhard.Hube@hki-jena.de

**Keywords:** MAP kinase, invasion, cell wall, epithelium

## Abstract

*Candida albicans* is an important human fungal pathogen responsible for tens of millions of infections as well as hundreds of thousands of severe life-threatening infections each year. MAP kinase (MAPK) signal transduction pathways facilitate the sensing and adaptation to external stimuli and control the expression of key virulence factors such as the yeast-to-hypha transition, the biogenesis of the cell wall, and the interaction with the host. In the present study, we have combined molecular approaches and infection biology to analyse the role of *C. albicans* MAPK pathways during an epithelial invasion. Hog1 was found to be important for adhesion to abiotic surfaces but was dispensable for damage to epithelial cells. The Mkc1 cell wall integrity (CWI) and Cek1 pathways, on the other hand, were both required for oral epithelial damage. Analysis of the ability to penetrate nutrient-rich semi-solid media revealed a cooperative role for Cek1 and Mkc1 in this process. Finally, *cek2Δ* (as well as *cek1Δ*) but not *mkc1Δ* or *hog1Δ* mutants, exhibited elevated β-glucan unmasking as revealed by immunofluorescence studies. Therefore, the four MAPK pathways play distinct roles in adhesion, epithelial damage, invasion and cell wall remodelling that may contribute to the pathogenicity of *C. albicans*.

## 1. Introduction

*C. albicans* is a dimorphic fungus that forms part of the microbiota of healthy individuals but is able to cause different infections in humans, which are especially severe among immunocompromised individuals. *C. albicans* is the prevailing *Candida* spp. causing superficial infections and for this to occur, the fungus needs to adhere to, invade, and damage host tissues. Adhesion is mediated by surface adhesins that either covalently or non-covalently interact with receptors on the surface of host cells [1], while invasion requires penetration into the host tissues facilitating dissemination [2]; both processes are unequivocally associated with pathogenicity. *C. albicans* are able to invade nonphagocytic epithelial and endothelial cells either inducing their endocytosis or via active penetration [3,4]. Endocytosis can be induced by thimerosal or UV-killed *C. albicans* hyphal cells and it is mediated by interactions between the fungal invasins Als3 and Ssa1 and host E-cadherin [4,5], while active penetration requires living *C. albicans*. The ability to invade and damage host tissues has been associated with different hydrolytic enzymes such as proteases (i.e., aspartyl proteases of the Sap family) and lipases [6] and several other pathogenicity factors play different roles during infection [7]. More recently it was discovered that epithelial damage is mediated by the cytolytic toxin candidalysin [8].

Although *C. albicans* readily colonizes the human body, this niche is not a constant and protected environment and fungal cells are exposed to a series of conditions that challenge their survival. Osmotic and oxidative stress, oxygen and nutrient availability can significantly change in different locations and require the fungus to respond to them. Signal transduction pathways involving MAPKs are crucial mechanisms to trigger these adaptations. They are formed by a core of three kinases (MAPK, MAPKK and MAPKKK) that become activated by upstream membrane-associated proteins; in turn, they phosphorylate other proteins, mostly transcription factors that regulate the expression of specific genes. Four MAPK pathways have been reported in *C. albicans* [9,10]. The cell wall integrity pathway mediated by the Mkc1 MAPK is mainly involved in cell wall construction [11] but also invasion [12]. The high osmolarity glycerol pathway MAPK Hog1 mediates adaptation to osmotic and oxidative stress through the synthesis of glycerol [13] and activation of oxidative defences [14,15] but is also involved in morphogenesis, as *hog1Δ* mutants exhibit enhanced filamentation (see [16,17] for recent reviews). Both Cek1 and Cek2 have been implicated in mating [18] and Cek1 also plays a role in cell wall remodelling [19]. The Cek1 pathway has been also involved in the evasion of the immune system since *cek1Δ* mutants expose more β-1,3 glucan and α-1,2 and β-1,2-mannosides on the cell surface [20]. This leads to altered recognition by innate immune cells through Dectin-1 and galectin-3 receptors and, therefore, to enhanced phagocytosis [19,20]. In addition, MAPK pathways are involved in other relevant physiological processes such as quorum sensing, biofilm formation or filamentation [10]. These multiple physiological roles may explain why some strains defective in MAPK-signalling are less virulent in mouse models of systemic infection [21,22,23]. The HOG pathway is also required for commensal colonisation of the mouse gastrointestinal tract [24]. Given the importance of MAPK signalling in *C. albicans* physiology and pathogenicity, we have tested the ability of MAPK single and double mutants for their ability to adhere to abiotic surfaces, to invade agar and host epithelial cells, to damage these cells, as well as β-1,3 glucan exposure of their cell walls.

## 2. Materials and Methods

### 2.1. Strains and Growth Conditions

Stains used are listed in Table 1. *C. albicans* was routinely grown at 37 °C in YPD medium unless otherwise stated. Culture growth in liquid medium was assessed through the measurement of absorbance at 600 nm in a Beckman DU 640 spectrophotometer. Strains were short-term stored at 4 °C in solid media and long-time storage of *C. albicans* strains was made at −80 °C in 50% (*v/v*) glycerol.

### 2.2. Adhesion Assays

To measure the adhesion to polystyrene, *C. albicans* exponentially growing cells were allowed to interact in DMEM (Dulbecco’s Modified Eagle Medium) medium in 24-wells plates and the fraction of adherent and not adherent cells were recovered at different times (0, 30, 60, 120 and 240 min). Non-dherent cells were obtained by removing the supernatant; PBS was then added to each well, gently washed and this supernatant was again removed; both supernatants were mixed. Adhered cells were obtained by carefully scratching the surface after supernatant removal with a glass rod in an appropriate volume of PBS. Both fractions were spotted on YPD-agar. The % of both fractions was obtained from CFU counts as (CFUs adhered fraction/(CFUs adhered fraction + CFUs supernatant fraction)) × 100. The adhesion of *C. albicans* cells to the plastic surface of μ-Slide VI (ibidi), was assessed through a circulatory flow model system developed by Wilson and Hube [30]. The media and the perfusion set were equilibrated overnight at 37 °C and 5% CO_2_. Eleven millilitres of DMEM without FBS was added to the perfusion set reservoirs and air bubbles were removed. The perfusion set was then joined to the μ-Slide (channel 1) via Luer connections. *C. albicans* yeast cells (from 30 °C overnight YPD cultures, washed three times with PBS) were added to the reservoirs to a final concentration of 5 × 10^5^ cells/mL and incubated at 37 °C and 5% CO_2_ under flow conditions. After 30 min incubation, the Luer connections were switched to channel 2 and flow was allowed for another 30 min. The channels were immediately flush washed three times with PBS and flush-fixed three times with Histofix^®^ (Roth). Adhesion assessment was achieved through microscopic analyses of 80 different fields for cells adherent to the slide and germ tube quantification was achieved through the microscopic analysis of 200 adherent cells per strain. The assay was performed twice for each strain.

### 2.3. Invasion Assays

Monolayers of oral epithelial cells (TR146) were grown on 15 mm diameter glass coverslips. The monolayers were infected with 10^4^ *C. albicans* cells and allowed to interact for 3 h. After this period, the medium covering the cells was aspirated and monolayers were rinsed three times with PBS to remove fungal cells that were not associated with epithelial cells. The samples were then fixed with Histofix^®^ and invasion of adherent cells was quantified through fluorescent microscopy using a differential staining procedure. All fungal cells that remained adherent to the surface of the epithelial cells were stained for 1 h with Alexa Fluor 488 conjugate of succinylated concanavalin A (Con A) (Invitrogen) which stained only the extracellular, non-invaded fungal elements. After rinsing with PBS, epithelial cells were permeabilized with 0.5% Triton X-100 in PBS for 5–10 min. Next, complete fungal cells (i.e., invaded and non-invaded) were stained with calcofluor white. The coverslips were then rinsed with water, mounted inverted onto slides, and the stained cells were visualized with epifluorescence (Leica DM5500B) using filter sets to detect Alexa Fluor 488 and calcofluor. The percentage of invading *C. albicans* cells was determined by dividing the number of internalized cells by the total number of adherent cells to the epithelial monolayer. At least 100 fungal cells were analysed on each coverslip and all experiments were performed in duplicates on at least two separate occasions. Images were taken with a Leica Digital Camera DFC360 FX.

To assess cells’ ability to invade solid media, 10^6^ cells of different strains were inoculated onto YPD agar media and incubated at 30 °C for up to seven days. At 2 days, the colonies were gently washed for invasion assessment. At 5 and 7 days, transversal cuts were made in the agar. Colonies were photographed after cutting the agar and laying it on its side to observe the invading cells through a magnifier WILD Heerbrugg M5-46860 coupled to a digital camera Panasonic Lumix DMC-G1K lens kit. Pictures were taken likewise for each experiment and processed using Adobe Photoshop CS5.

### 2.4. Damage Assay

Epithelial cell damage caused by different *C. albicans* strains during interaction with TR146 cells was determined by the release of lactate dehydrogenase (LDH) into the surrounding medium, following 24 h uninterrupted co-incubation. TR146 monolayers were grown until confluence in 96 well culture plates and infected with 2 × 10^4^ cells in DMEM without FBS (previously washed with PBS) and placed in a humidified incubator (37 °C and 5% CO_2_) for 24 h. For control samples, TR146 cells were incubated with DMEM medium only. After 24 h, 0.2%Triton X-100 was added to uninfected high control wells to induce epithelial lysis, and the extracellular LDH release into the medium of all samples was measured spectrophotometrically at 492 nm using the Cytotoxicity Detection Kit (LDH) from Roche Applied Science according to the manufacturer’s instructions. The percentage of damage of epithelial cells infected with *C. albicans* was calculated comparing to the experimental LDH release by Triton X-100 treatment (high control—100% lysis). Damage experiments were performed at least twice in triplicate for all strains.

### 2.5. Glucan Exposure Analysis

β-glucan exposure was determined by immunofluorescence as described [20]. Briefly, either live or heat-killed (HK) stationary phase cells (24 overnight growth in YPD medium) were washed in PBS buffer and incubated with anti-β-(1,3)-glucan MAb (Biosupplies) followed by labelling to a secondary antibody conjugated to Alexa288 prior to the analysis in a Guava (Millipore) flow cytometer.

## 3. Results

### 3.1. Strains Lacking the Hog1 MAPK Display Adhesion Defects to Abiotic Surfaces

Microbial adhesion represents the initial stage of interaction during both commensal and pathogenic relationships [2,31] and is critical in host-pathogen interactions. We, therefore, analysed the behaviour of *C. albicans* cells deficient in MAPK signalling in their ability to adhere to an abiotic surface (polystyrene) using two different models.

**Figure 1:**
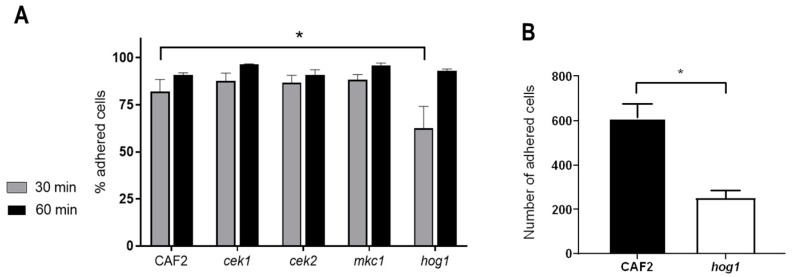
Adhesion to different surfaces of *C. albicans* strains defective in MAP kinases (**A**) *C. albicans* cells growing exponentially at 30 °C were counted and inoculated into 24-wells polystyrene plates. The assay was performed in DMEM medium at 37 °C and 5% CO_2_ during 30 and 60 min. Adhered and non-adherent *C. albicans* cells were recovered and spread on YPD plates for CFUs counting. Data are the mean of four independent experiments and the error bars stand for SEM (Standard Error of the Mean). * *p* < 0.05. (**B**) Number of *C. albicans* cells adhered to plastic exposed to a continuous flow. *C. albicans* cells were inoculated in DMEM medium to a final concentration of 5 × 10^5^ cells/mL and circulated within an ibidi flow system at 37 °C and 5% CO_2_. Quantification of the cells was done microscopically analysing 80 different fields. The experiments were performed twice for each strain and the errors bars represent the SEM.

Cellular adhesion to abiotic plastic surfaces such as catheters and artificial joints is particularly important in *C. albicans* pathogenicity as it leads to the formation of biofilms on these devices [32]. Adhesion to polystyrene was performed using conventional cell culture plastic plates. The *hog1Δ* mutant strain adhered less to the polystyrene surface than the wild-type under static conditions at 30 min, showing 62.5% ± 11.7% adhesion compared to 82% ± 6.5% for CAF2 (*p* < 0.05, one-way ANOVA). Deletion of the other three MAP kinases had no impact on adhesion at this time At extended interaction periods (60 min), all analysed strains were able to adhere to the polystyrene surface at levels similar to the wild-type (Figure 1A).

Therefore, Hog1 plays a role in adhesion under static conditions. However, in many anatomical niches (the circulatory system, for example), *C. albicans* encounter abiotic surfaces under conditions of shear stress and flow. We, therefore, tested strains for their capacity to adhere to a unidirectional flow model [30]. We quantified adhesion to the plastic surface following 60 min of flow conditions: wild-type cells exhibited robust adhesion (Figure 1B) in parallel with the appearance of germ tubes by adherent cells (not shown). Differences in adhesion of *cek1Δ* and *mkc1Δ* strains were not found statistically significant (data not shown). However, the *hog1Δ* mutant exhibited significantly less adhesion compared to the wt at 60 min (251 ± 35 versus 613 ± 63 (Figure 1B); this was not a defect caused by altered germination, as these values were similar to the wt (51.5% for *hog1Δ* mutants and 49.8% for the wt). These differences in adhesion were also confirmed through the quantification of the unadhered cells that remained in the circulating medium at the end of the experiment.

These data demonstrate that Hog1 plays a role in adhesion of *C. albicans* cells to abiotic surfaces at early stages, that the kinase plays an important role in adhesion under circulatory flow and that these differences are not due to a different germination efficiency.

### 3.2. Hog1 Negatively Regulate Agar Invasion While Cek1/Cek2-Mediated Pathways Participate in Oral Epithelium Invasion

Hog1, Mkc1 and Cek1 have each been demonstrated to be important for virulence in mouse models of systemic candidiasis [21,22,23]. We, therefore, questioned whether these *C. albicans* MAPK pathways also played roles in superficial infections.

**Figure 2:**
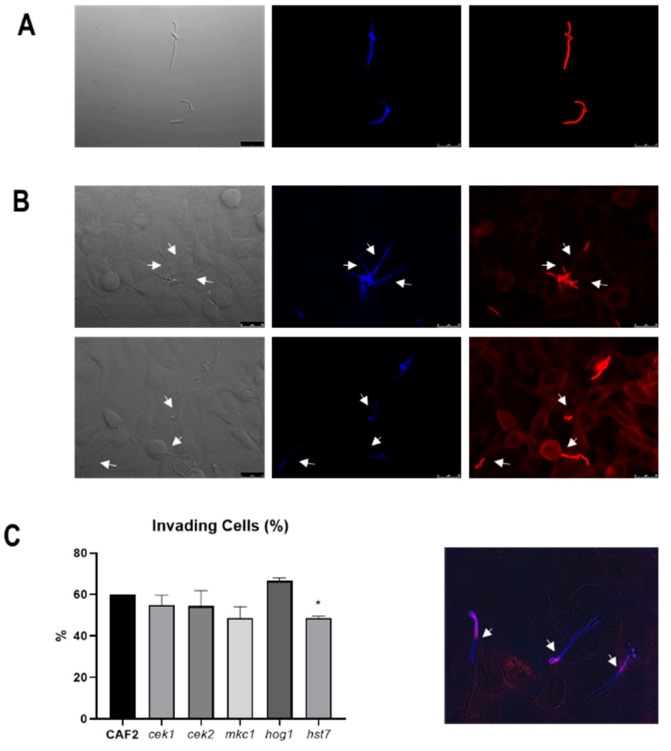
Epithelial invasion assay by *C. albicans* strains. (**A**) Staining control of *C. albicans* cells without epithelia treated with calcofluor white (seen in blue) and Alexa Fluor 488 conjugate of succinylated concanavalin A, in red. (**B**) Representative fluorescence microscopic images of *C. albicans* invading epithelial cells. The white arrows indicate the point where hyphae breach the epithelium and Con A staining disappears (better appreciated in the merged image shown in the lower panel). Bars indicate 25 µm. (**C**) Quantification of *C. albicans* cells invading the epithelium. Percentages are related to the starting amount of cells and error bars represent the standard deviation of the mean from three independent experiments. At least 100 fungal cells were analysed on each experiment. * *p* < 0.05.

We first tested the invasion of MAPK defective mutants using a TR146 oral epithelial monolayer infection model coupled with differential fluorescent staining (Figure 2). Following 3 h infection, approximately 60% of wild-type cells had invaded epithelium. Invasion was similar for *hog1Δ* (66.6% ± 1.5) *cek1Δ* (54.9 ± 4.7% (*p* = 0.25), *cek2Δ* (54.4 ± 7.5% (*p* = 0.41) and *mkc1Δ* (48.5 ± 5.5% (*p* = 0.057) mutants (Figure 2C).

The terminal MAPKs Cek1 and Cek2 are both regulated by the MAPKK Hst7. We, therefore, analysed the invasive ability of strains defective in the *HST7* gene. Interestingly, the absence of Hst7 significantly reduced invasion to only 48.7 ± 0.62% (*p* = 0.003). We conclude that the *HST7* MAPKK (Cek1/Cek2-mediated) pathway plays a role in epithelial cell invasion in this model.

**Figure 3:**
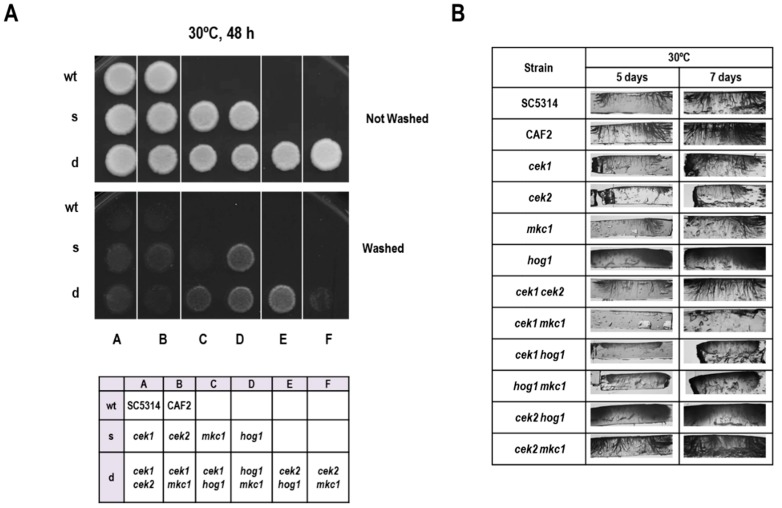
Analysis of invasion of solid media. (**A**) 10^6^ cells from overnight grown cultures were spotted on YPD agar plates and incubated for 48 h at 30 °C. The plates were gently washed to allow the analysis of colonies’ invasion (lower panel). A panel showing the position of strains is shown below. (**B**) 10^6^ cells from overnight grown cultures were spotted on YPD agar plates and incubated for 5 or 7 days at 30 °C. The plates were gently washed and transversal cuts made for the observation of the invading filaments.

The role of MAPK pathways during the invasion was also assessed on solid media. For this purpose, patches of cells were allowed to grow on YPD solid medium and invasion was tested by carefully washing the surface after 48 h of growth at 30 °C. As shown in Figure 3A, *hog1Δ* cells were hyper invasive in these conditions, as reported previously [21]. In order to show a putative role of other MAPKs during the invasion of *hog1Δ* cells, we tested a set of mutants altered in all combinations of MAPKs. Deletion of *CEK1* (but neither *CEK2* nor *MKC1*) in this background resulted in partial suppression of the phenotype, as the *hog1Δ cek1Δ* double mutants invaded the agar to a lesser extent than *hog1Δ*. The rest of the mutants showed no visible difference in phenotype compared to the wild type. These surface invasion observations were then confirmed by performing transversal cuts of the agar (Figure 3B): the *hog1Δ cek1Δ* double mutant showed shorter hyphal penetration. Interestingly, the *cek1Δ mkc1Δ* double mutant (but not *cek2Δ mkc1Δ*) was almost completely unable to penetrate the agar. Collectively, these results indicate that Cek1 and, to a lesser extent Mkc1, promote agar invasion.

### 3.3. Mkc1 and Cek1 Mediate Oral Epithelial Damage

Epithelial damage is a key characteristic of *C. albicans* pathogenesis and occurs when hyphae invade deep into or through host cells and relate to adherence and invasion. We analysed the role of MAPK signalling pathways in epithelial damage by using our strains defective in one or two MAP kinases.

**Figure 4:**
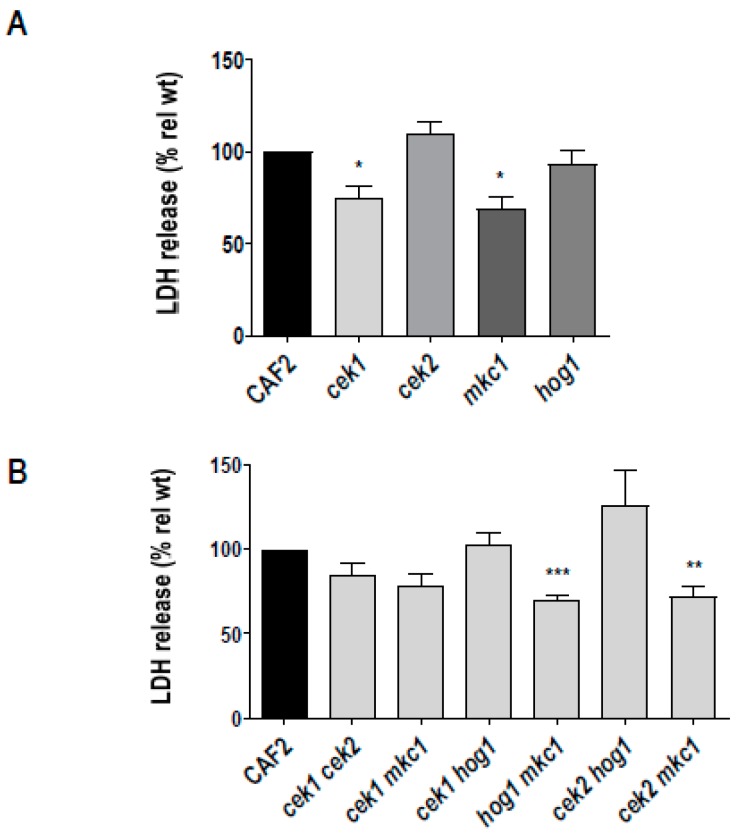
Damage of oral epithelial cells by *C. albicans* MAP kinase defective strains. 2 × 10^4^ *C. albicans* cells were incubated with a TR146 cell line monolayer for 24 h. Damage of the epithelial cells was quantified by measuring the release of lactate dehydrogenase (LDH) into the surrounding medium. Damage for each strain was normalised to the wild type damage (CAF2). Errors bars represent the SEM of at least three independent experiments., * *p* < 0.05, ** *p* < 0.01, *** *p* < 0.005.

*C. albicans* cells were used to infect a confluent oral epithelial cell monolayer (TR146) for 24 h and damage assessed by measuring epithelial lactate dehydrogenase (LDH) release. Deletion of *CEK1* or *MKC1* reduced damage by 25% and 31%, respectively, whilst *cek2Δ* and *hog1Δ* exhibited damage levels comparable to the wild type (Figure 4A). Mkc1 has been previously implicated in epithelial damage [3] and here, we additionally demonstrate a role for the Cek1-mediated pathway in this process.

In order to reveal the possible cooperative role of MAPK pathways in epithelial damage, we performed LDH assays using double MAPK mutants (Figure 4B). Figure 4B shows that the *hog1Δ mkc1Δ* and *cek2Δ mkc1Δ* mutants exhibited significantly reduced epithelial damage, in line with the reduced damage of the *mkc1Δ* single mutant. The *cek1Δ cek2Δ* and *cek1Δ mkc1Δ* strains were also reduced, but these effects were not statistically significant. Interestingly, simultaneous deletion of *CEK1* and *HOG1* completely rescued the epithelial damage defect of *cek1Δ* and the *cek2Δ hog1Δ* caused elevated levels of damage. This indicated that, in the absence of Cek1 or Cek2 signalling, Hog1 may actually dampen epithelial damage—this may be due to the fact that Hog1 can negatively regulate filamentous growth in *C. albicans*.

### 3.4. Cek2 Is Involved in Glucan Masking

**Figure 5:**
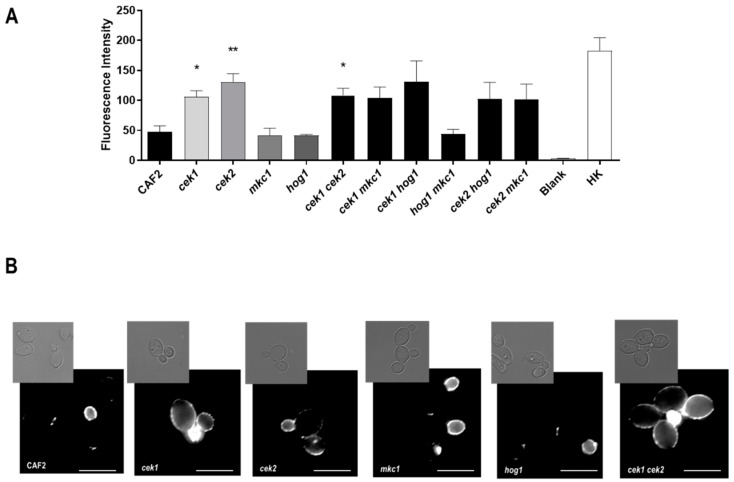
Analysis of β-glucan exposure in MAPK mutants. (**A**) Mean values of the fluorescence intensity for each strain (arbitrary units) and the standard error of the mean (SEM) from three independent experiments are shown. Heat-killed cells (HK), used as positive control Blank refers to cells treated only with the secondary antibody. * *p* < 0.05, ** *p* < 0.01 (**B**) Fluorescence microscopy analysis of cells β glucan exposure. Representative pictures of fluorescence microscopy analysis Pictures were taken and processed likewise for brightness and contrast adjustments. Upper panels, brightfield; lower panels, immunofluorescence microscopy. The white bar represents 5 µm.

We next investigated the impact of *C. albicans* MAPKs on β-glucan exposure—a key cell wall component recognised by the immune system. Cells were grown to exponential phase in YPD, stained with anti-β-(1,3)-glucan antibodies and fluorescence intensity quantified by flow cytometry. As shown in Figure 5A, deletion of *HOG1* or *MKC1*, either alone or in combination, did not influence β-glucan exposure; in contrast, all seven strains lacking *CEK1* and/or *CEK2* exhibited strong (at least two-fold) increased β-glucan levels compared to the wild type and for *cek1Δ*, *cek2Δ* and *cek1Δ cek2Δ* this was statistically significant.

Fluorescence microscopy revealed that, as expected, β-glucan exposure was restricted to the daughter cell and bud scars of *CEK1*^+^ *CEK2*^+^ cells, with mother cells showing little or no exposure. In contrast, mutants lacking *CEK1* and/or *CEK2* exhibited a clear punctate labelling pattern on the surface of the mother as well as daughter cells (Figure 5B), consistent with the enhanced mean fluorescence signals measured flow cytometry analysis (Figure 5A). These results indicate that both Cek1 and Cek2 MAPKs modulate glucan exposure in the *C. albicans* cell wall.

## 4. Discussion

In this work, we have undertaken a systematic analysis of the contribution of the four *C. albicans* MAPKs to processes relevant during pathogenesis, such as adherence, invasion and damage to epithelial cells, using different epithelium models and abiotic surfaces, and cell wall architecture. Surprisingly, although the four analysed MAPKs have been reported to play a role in cell wall biogenesis [11,21,27], only the *hog1Δ* mutant displayed reduced adhesion. Under static conditions, this defect was observed at short (30 min) times, suggesting that the Hog1 pathway may be important for the early expression of factors involved in adhesion. Adhesion under flow was strongly reduced for the *hog1Δ* mutant. Interestingly, an earlier report showed that the hypha-specific cyclin Hgc1 was crucial for adhesion under flow conditions [30] and Hog1 has been implicated in the regulation of Hgc1 [33].

Epithelial invasion is triggered by filament formation and mutants defective in the cAMP/PKA pathway and the transcription factor Efg1 exhibit defective filamentation, do not adhere and invade less efficiently. None of the mutants defective in MAPK displayed defects in germination and all invaded epithelial cells similarly to the parental CAF2 strain. Slight increases in the percentage of invading cells were observed in the *hog1Δ* mutant, probably influenced by the repressive role in yeast-to-hypha [21,34]. However, the *hst7Δ* mutant showed a reduced percentage of invading cells. Because Hst7 is the MAPKK responsible for phosphorylating and activating both Cek1 and Cek2, this suggests that these MAPKs may be involved in epithelial invasion. Cek1 was firstly reported to be involved in invasive hyphal growth on Spider agar plates [23] and *MKC1* has been shown to mediate invasion of solid surfaces on agar minimal medium [12]. Here, we confirm these data using a different nutrient-rich solid medium and demonstrate that Cek2 is also involved in this process. Deletion of both *MKC1* and *CEK1* MAPKs delay the invasive phenotype and their effects are additive on YPD plates, pointing towards separate roles for both MAPKs in this process.

Although the *hog1Δ* mutant can filament more readily than wild type cells [21], invasion of *hog1Δ* into oral epithelial cells was only modestly higher than wild type. This may be because contact with epithelium, together with other environmental parameters associated with cell culture (37 °C, neutral pH, 5% CO_2_), are very strong stimulators of hypha formation in the wild type and likely largely bypass Hog1 hyphal-repression. Indeed, when tested under weaker hyphae-stimulating (YPD agar at 30 °C), invasion by *hog1Δ* was much more robust than that of wild type. Invasion of epithelial cells does not necessarily cause cell damage; lyses of the epithelial cells and tissue destruction have been shown to be caused in part by a secreted cytolytic toxin called candidalysin [8] and derived from the proteolytic digestion of Ece1. *ECE1* is highly expressed during hyphal formation [35] and its expression has been reported to be negatively regulated by Hog1 [15,36]. However, the lack of Hog1 increases *ECE1* expression in exponentially growing cells in YPD medium (not shown) and also enhances filamentation but had no effect on damage to epithelial cells; increased *ECE1* levels are, however, far away from those attained when *C. albicans* wild type cells contact the epithelium, in an environment of 37 °C, neutral pH and 5% CO_2_ that upregulate *ECE1* 20,000-fold [8]. Therefore, any negative effect Hog1 is having on *ECE1* expression in Figure 4 is likely not having any impact on damage.

We also show here that *MKC1* mediates damage to epithelial cells, confirming a previous work by Wachtler and co-workers [3] that analysed several mutant strains previously described to be attenuated during host-pathogen interactions. We have also identified a novel role for Cek1 in epithelial damage. Although we observed a significant reduction in epithelial damage for *cek1Δ* and *mkc1Δ*, it was quite a moderate decrease compared to other regulatory factors, such as those governing hypha formation—for example strains lacking Ras1 or Eed1 exhibit epithelial damage reduction of over 80% compared to wild type [3]. Yet all three of the MAPK mutants can exhibit almost complete avirulence in animal models of invasive. Together with our observations, this suggests that MAPK signalling is critical for systemic infection, but plays a relatively minor role or, in the case of Hog1, is dispensable for epithelial infection, under the conditions tested here.

We show that both Cek1 and Cek2 play important roles in β-glucan masking in *C. albicans*. Although Cek2 had historically been thought to only mediate mating, we had previously shown that this MAPK has a cryptic role in cell wall construction [27], as revealed by the sensitivity of *cek2Δ* to cell wall antifungals and becomes activated by the same stimuli as Cek1. Therefore, defective cell wall biogenesis may account for increased β-glucan exposure. Cek1 is known to play a role in glucan masking [20] via disorganisation of the external mannoprotein layer [19]. In parallel, hyperactivation of Cek1 also promotes β (1,3)-glucan unmasking by up-regulation of cell wall proteins [37] which improves immune recognition in vivo allowing fungal clearance. These apparently paradoxical observations are probably due to the need for extremely tight regulation of Cek1 activity in the cell [38].

In summary, our results indicate that the four MAPKs identified and described in *C. albicans* play minor contributions during adhesion and invasion but Cek1 and Mkc1 both significantly impact epithelial damage and that Cek1/Cek2 play important roles in β-glucan masking. Both are important properties governing *C. albicans* pathogenicity and immune evasion and, therefore, represent important potential antifungal targets to control candidiasis.

## Figures and Tables

**Table 1:** Strains of *C. albicans* used in this work.

Strain Name	Background Strain and Genotype	Source
SC5314	Clinical isolate	[25]
CAF2	*ura3::imm434/ura3::imm434-URA3*	[26]
CAI4	*ura3::imm434/ura3::imm434*	[26]
*cek1Δ*	[CAI4] *cek1::hisG-URA3-hisG/cek1::hisG*	[23]
*cek2Δ*	[CAI4] *cek2::cat-URA3-cat/cek2::cat*	[27]
*mkc1Δ*	[CAI4] *mkc1::hisG-URA3-hisG/mkc1::hisG*	[11]
*hog1Δ*	[CAI4] *hog1::hisG/hog1::hisG-URA3-hisG*	[24]
*cek1Δ cek2Δ*	[CAI4] *cek1::hisG/cek1::hisG cek2::cat-URA3-cat/cek2::cat*	[27]
*cek1Δ mkc1Δ*	[CAI4] *cek1::hisG/cek1::hisG mkc1::hisG-URA3-hisG/mkc1::hisG*	[27]
*cek1Δ hog1Δ*	[CAI4] *cek1::hisG/cek1::hisG hog1::hisG/hog1::hisG* *ARD1/ard1::FRT SAP2pr-FLPURA3*	[28]
*hog1Δ mkc1Δ*	[CAI4] *hog1::hisG/hog1::hisG* *mkc1::hisG-URA3-hisG/mkc1::hisG*	[27]
*cek2Δ hog1Δ*	[CAI4] *cek2::cat/cek2::cat hog1::hisG-URA3-hisG/hog1::hisG*	[27]
*cek2Δ mkc1Δ*	[CAI4] *cek2::cat/cek2::cat mkc1::hisG-URA3-hisG/mkc1::hisG*	[27]
*hst7Δ*	[CAI4] *hst7::hisG-URA3-hisG/hst7::hisG*	[29]
